# Esophageal achalasia in an adolescent in Central Africa: a case report

**DOI:** 10.11604/pamj.2021.40.155.28211

**Published:** 2021-11-16

**Authors:** Trésor Kibangula Kasanga, Manix Ilunga Banza, Florent Tshibwid A Zeng, Augustin Kibonge Mukakala, Jonas Tshilolo-Yona, Williams Kalala Mpumbwa, Anatole Nyembwe Mbuyi, Olivier Tshiteya Kabeya, Sébastien Mbuyi-Musanzayi

**Affiliations:** 1 Department of Surgery, University Clinics of Lubumbashi, University of Lubumbashi, Lubumbashi, Democratic Republic of Congo,; 2 Service of Surgery, Medicare Hospital, Lubumbashi, Democratic Republic of Congo,; 3 Service of Surgery, Medicare Hospital, Lubumbashi, Democratic Republic of Congo

**Keywords:** Esophageal achalasia, child, open Heller esophago-cardiomyotomy, case report

## Abstract

Esophageal Achalasia has rarely been reported in sub-Saharan Africa. We report a case of a 12 years old boy who has been diagnosed after experiencing dysphagia for a year and progressive wasting. Esophagogram was the only exploration available in our settings and showed classical features. He underwent a Heller esophago-cardiomyotomy with Toupet fundoplication. Postoperative period was unremarkable and BMI normalized for age and sex on the sixth postoperative month. In low settings, history is a key step which lead to clinical suspicion as esophagogram is often the only available exploration to confirm the diagnosis.

## Introduction

Esophageal achalasia (EA) is a primary motor disorder of the esophagus. It has been first described by Sir Thomas Willis in 1672 [1]. This condition is mainly found in adult patients, hence its frequency among children, is very rare. Its aetiology is not clearly elucidated, however, the idea of EA to be an autoimmune disease occurring in a certain genetic background and triggered by several factors is commonly admitted [2]. Patients present with a wide range of symptoms, depending on age and stage of the disease. Since the cause is still not fully understood, the treatment remains symptomatic, using pharmacological, endoscopic and surgical means. We present the case of a 12 years old male adolescent brought in our service at the University clinic of Lubumbashi. We discuss epidemiology, pathogeny, clinical presentation, investigations, surgical treatment and outcomes of EA in paediatric population.

## Patient and observation

**Patient information:** we received a 12 years old male adolescent, brought to our service for dysphagia for both liquids and solids, which started a year ago. At its beginning, the patient could improve the swallowing by drinking more water during meals and by having small bites. Dysphagia progressively worsened as in following months, appeared regurgitation following meals after some hours, made of undigested food of the latest meals. In the last 6 months, parents describe a progressive wasting for which they have consulted a medical centre where no clear solution has been found. Then the patient started experiencing a retrosternal discomfort, described as heartburn as well as sialorrhea. As symptoms were worsening, parents decided to consult at our hospital. The patient has normal infancy and childhood periods, without any specific swallowing complaint. There was no history of caustic ingestion and the patient has never travelled in South America or any other region endemic to Trypanosoma cruzi. He had no herpetic infection earlier, neither a severe bacterial infection. Such disorder has never been noticed in his siblings, neither in his father nor mother´s family.

**Timeline:** first symptoms were experienced by the child in October 2019 as dysphagia, which progressively worsened from then to April 2020. From May 2020, parents report wasting for which they attended a medical centre in June 2020, with no solution provided. Additional symptoms (heartburn and sialorrhea) appeared in September 2020, which lead parents to attend our hospital on 07 October 2020, where diagnosis has been retained on 8 October 2020 and surgery performed a day later.

**Figure 1:**
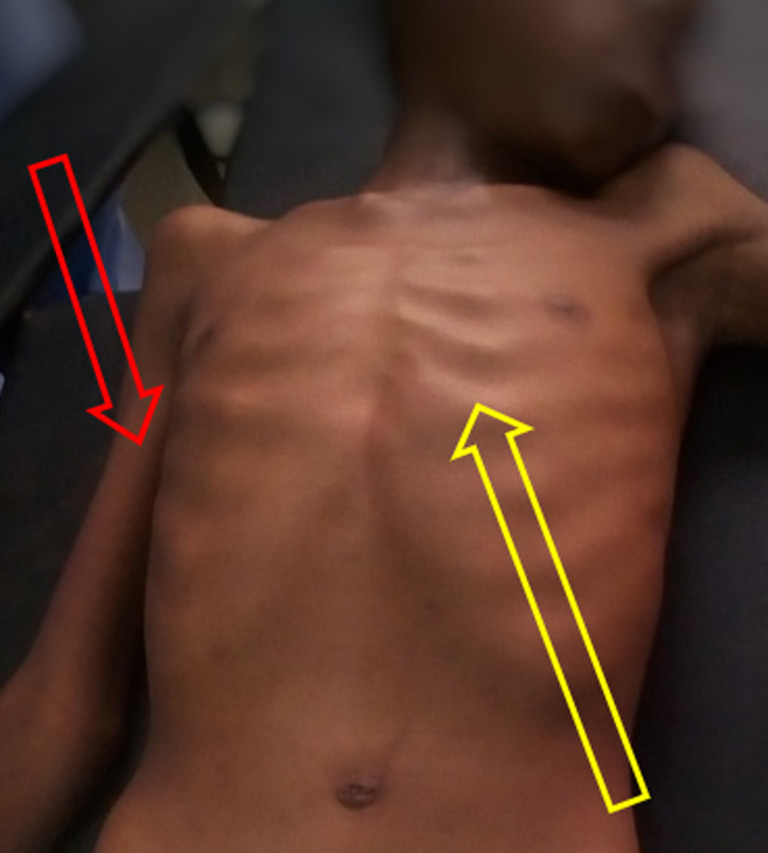
picture showing the wasted patient; prominent ribs (yellow arrow) and thin limbs (red arrow)

**Clinical finding:** on physical examination, the patient was conscious. He was wasted and with moderate dehydration. Body temperature was normal, he had polypnea and tachycardia, his blood pressure was normal. He weighed 26 Kg and had a height of 124 Cm, his body mass index (BMI) was 11.9 Kg/m^2^, (Figure 1). The rest of the examination was not contributive. There were neither alacrimia, nor features of adrenal insufficiency, nor vitiligo or deafness.

**Figure 2:**
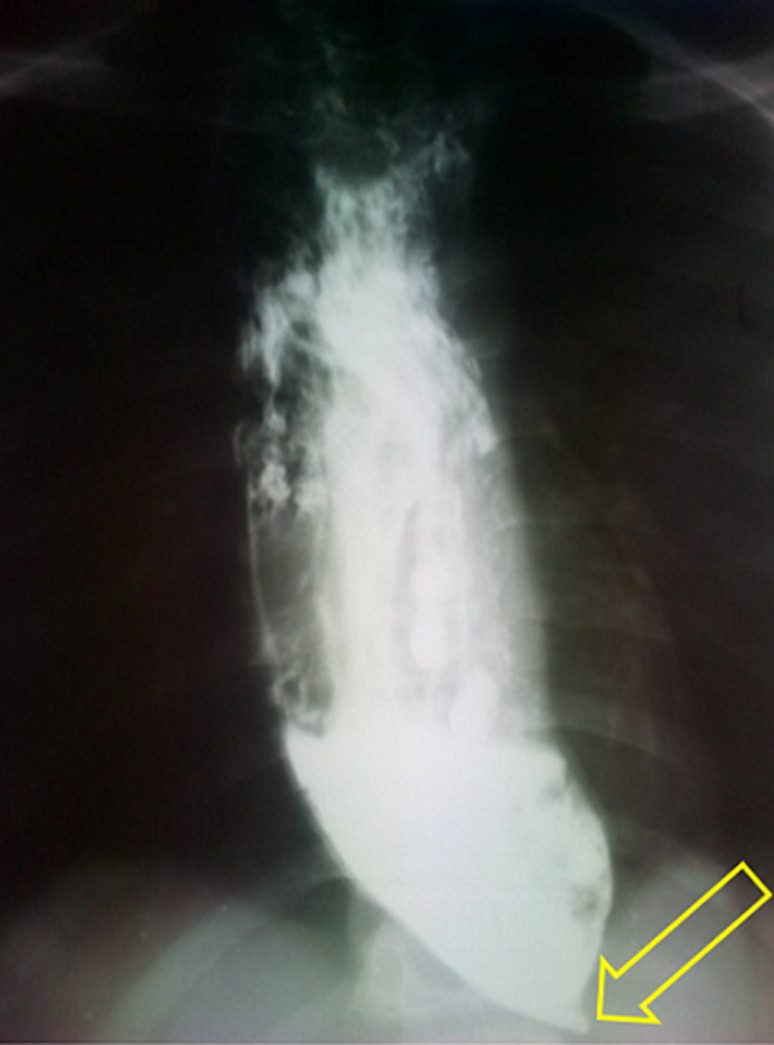
esophagogram showing the classic bird´s beak of achalasia (yellow arrow) and dilatated esophagus above

**Diagnostic assessment:** investigations included blood tests: glucose, natrium and potassium, which shown moderate hypoglycaemia. Imaging included an esophagogram which showed a dilated esophagus, with stasis of the contrast meal and a progressive narrowing picturing the classic bird´s beak of achalasia cardia (Figure 2). Due to lack of equipment and financial constraint, no further investigation could be done, manometry and endoscopy, respectively.

**Figure 3:**
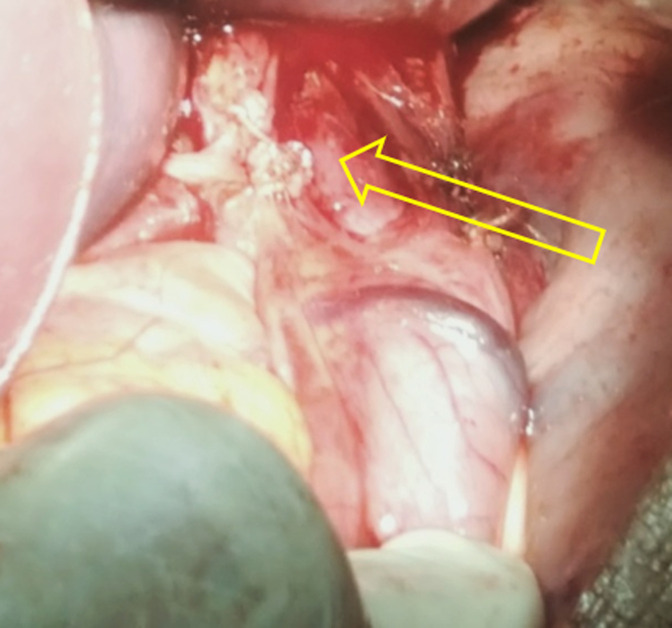
intraoperative image of the cardiomyotomy extending from the esophagus to the upper part of the anterior side of the stomach; note the bulging mucosa (yellow arrow)

**Therapeutic intervention:** at our service of emergency, rehydration has been started as well as parenteral nutrition. After two days of reanimation, the patient underwent a median sus umbilical laparotomy. A Heller esophago-cardiomyotomy has been performed with a five centimetre incision, running from the lower esophagus to the cardia, on the anterior face of the stomach. A Toupet fundoplication has been added as an antireflux procedure, with stitches placed at esophago-cardiomyotomy´s edges (Figure 3).

**Follow-up and outcomes:** paracetamol has been used as postoperative analgesic (15 mg/Kg, qid), what about antibiotic? and oral feeding started 24 hours after surgery. The postoperative period has been smooth and the patient has been released on postoperative day five. The patient has been seen on postoperative day 10 with no major complaint and then, on the third and sixth months after surgery. All symptoms have remarkably regressed since postoperative day five. At the latest follow-up (6 months after surgery), the patient doubled his weight, going from 24 to 48 Kg. The patient will be yearly followed and during his second decade, endoscopic screening for esophageal carcinoma will be started.

## Discussion

Strengths of our approach include the rapid and adapted diagnosis and management of our patient, with an appropriate antireflux procedure. Limits are the lack of further investigations due to reasons cited earlier.

Esophageal achalasia, also called achalasia cardia or cardiospasm is a functional disorder which has 3 main features: (a) absence of esophageal body peristalsis, (b) incomplete or delayed or absent relaxation of the lower esophageal sphincter (LES) and (c) increased resting pressure of the LES [3]. It has been identified more than nearly 350 years ago by Sir Thomas Willis. In 1882, Von Mikulicz used the term “cardiospasm&#8221 and was the first to think the problem would be functional. Ernest Heller performed the first surgical treatment in 1913 as a double myotomy, on both anterior and posterior sides of the esophagus. We owe the term ‘achalasia’ to Hurt and Rake who remarked the inability of the LES to relax [1].

EA is a rare disease in children, only four to five percent of all reported cases are children [4]. Several large population-based studies report an increasing incidence in children, ranging from 0.11 to 0.18 for 100,000 children per year. It generally more affects boys [3, 5]. Most cases are diagnosed between 7 and 13 years old, rarely in preschool age, as infant represent less than six percent of all diagnosed children [2].

The cause of this condition is still unclear. The most admitted theory is that EA is an acquired autoimmune disease with a genetic background and triggered by various infections: viral (Herpes), bacterial or parasitic (T. cruzi) [2]. The autoimmune postulate is supported by findings in the LES (myenteric inflammation and serum antibodies in the myenteric plexus) and increased frequency of Class II histocompatibility antigens in achalasia patients [6]. Association with genetic syndromes such as Allgrove (Triple A), Central Cerebral Hypoventilation and Rozycki, reinforces the idea of a genetic role [2]. Inflammation leads to morphology modification and number reduction of ganglia, especially NO-releasing neurons in the LES. Several authors reported reduced VIP immunoactivity in nerve fibers in EA [3]. Reduced NO and VIP results in an imbalance between excitatory and inhibitory neurotransmitters, ending in a hypertensive non-relaxed LES [1].

Clinical findings depend on age. In infants, regurgitation is common, occurring a few hours after meals. Respiratory symptoms are also common, these include wheezing and nighttime cough [1, 5]. In older children, the most common symptom is dysphagia, which is progressive for both solid and liquid. Other symptoms include pain on swallowing, regurgitation, retrosternal pain, sialorrhea, respiratory symptoms and failure to thrive, depending on the duration of dysphagia [1, 5].

Different investigations are possible in case of EA. Chest X-ray shows an esophageal air fluid level without gastric bubble on erect position. A widened mediastinum or pneumonia signs can also be seen [1, 5]. Esophagogram depicts a dilatated esophagus, with lack of peristalsis on its body, with progressive narrowing realizing the classic bird´s beak sign or rat tail deformity. There is also a failure of the contrast product to pass [6, 7]. In African and other Low and medium incoming countries (LMICs), Esophagogram is an important study since many centers lack manometry [5]. Upper endoscopy visualizes a dilatated esophagus with retention of chewed-up food and secretions, without any gastroesophageal obstruction. Due to yeast infection, certain degree of inflammation can be objectivated. This study also helps to exclude an organic cause of obstruction [5, 7]. Manometry is the gold standard for diagnosing EA. It reveals the three criteria which define the disease: aperistalsis of the esophageal body, absent or delayed relaxation of LES and its elevated resting pressure, more than 20±10 mmHg [8].

In adults, many possibilities of treatment of EA have been explored. They comprise pharmacological, endoscopic and surgical means. The two earlier have shown their limit in paediatric population/Pharmacological treatment, made of calcium channel blockers, nitrates or phosphodiesterase-5 inhibitors must be prescribed for long duration and has many side effects. Endoscopic options include dilatation and botulinum toxin injection. Dilatation has a long-term success of 40 to 60% and only 25% of patients showed good response [5, 9]. This is not exploitable in children as a good long-term success is mandatory. The same situation is encountered with botulinum toxin injection, for which 50% of patients need a reinjection after a year [3].

For paediatric population, surgery is the most appropriate treatment. Since its description by Heller more than a century ago, cardiomyotomy remains the classical procedure, however it is realized only on the anterior side nowadays as a single incision has shown its efficacity. Presently, the lower esophagus can be reached via transesophageal, thoracic or abdominal approaches, by using endoscopic, open or laparoscopic procedures. Additionally, an antireflux procedure is done to minimize risks of gastroesophageal reflux after the procedure. Dor, Thal and Toupet fundoplications are the most preferred methods comparing to Nissen´s fundoplication. The two first have the advantage to not weaken anatomical antireflux structures by avoiding posterior dissection. As they are anterior procedures, their second advantage is to provide a coverage of the mucosa, which reduces risk of perforation. The main advantage of Toupet fundoplication is to maintain the cardiomyotomy opened, since stitches are placed on its edges. Nissen´s procedure is avoided mainly due to dysphagia resulting from a 360° wrapping [8]. Peroral endoscopic myotomy is an innovative approach introduced more than a decade now. Its results are similar to those of laparoscopic approach, but there is a lack of long-term follow-up report to assess its long-term outcomes [8].

Outcomes are favourable, without any complication in 80% [6]. The most feared complication is perforation. It is the reason why an esophagogram should be performed after surgery to roll out a leakage. Persistence of symptoms after surgery can be due to an incomplete esophago-cardiomyotomy. In this case, a new procedure should be performed on the posterior side of the esophagus [1]. Due to the well-established association between EA and esophageal neoplasms, a long-term follow-up is mandatory. However, no consensus has been reached on when to start the follow-up, but some authors suggest the second decade [10].

EA is scarce in paediatric population. High clinical suspicion is required as history often provide key elements for the diagnosis. In our condition, esophagogram was the only available exploration, it is the case in many other LMICs. Heller´s esophago-cardiomyotomy plus an antireflux procedure is the standard treatment in children and in our conditions, we performed it via an open abdominal approach. A long-term follow-up has been planned.

## Conclusion

In low settings, history is a key step which lead to clinical suspicion as esophagogram is often the only available exploration to confirm the diagnosis.
